# Sampling for Microsatellite-Based Population Genetic Studies: 25 to 30 Individuals per Population Is Enough to Accurately Estimate Allele Frequencies

**DOI:** 10.1371/journal.pone.0045170

**Published:** 2012-09-12

**Authors:** Marie L. Hale, Theresa M. Burg, Tammy E. Steeves

**Affiliations:** 1 School of Biological Sciences, University of Canterbury, Christchurch, New Zealand; 2 Department of Biology, University of Lethbridge, Lethbridge, Canada; British Columbia Centre for Excellence in HIV/AIDS, Canada

## Abstract

One of the most common questions asked before starting a new population genetic study using microsatellite allele frequencies is “how many individuals do I need to sample from each population?” This question has previously been answered by addressing how many individuals are needed to detect all of the alleles present in a population (i.e. rarefaction based analyses). However, we argue that obtaining accurate allele frequencies and accurate estimates of diversity are much more important than detecting all of the alleles, given that very rare alleles (i.e. new mutations) are not very informative for assessing genetic diversity within a population or genetic structure among populations. Here we present a comparison of allele frequencies, expected heterozygosities and genetic distances between real and simulated populations by randomly subsampling 5–100 individuals from four empirical microsatellite genotype datasets (*Formica lugubris*, *Sciurus vulgaris*, *Thalassarche melanophris*, and *Himantopus novaezelandia*) to create 100 replicate datasets at each sample size. Despite differences in taxon (two birds, one mammal, one insect), population size, number of loci and polymorphism across loci, the degree of differences between simulated and empirical dataset allele frequencies, expected heterozygosities and pairwise F_ST_ values were almost identical among the four datasets at each sample size. Variability in allele frequency and expected heterozygosity among replicates decreased with increasing sample size, but these decreases were minimal above sample sizes of 25 to 30. Therefore, there appears to be little benefit in sampling more than 25 to 30 individuals per population for population genetic studies based on microsatellite allele frequencies.

## Introduction

One of the most common questions asked when embarking on a new population genetic study using microsatellite allele frequencies is: “how many individuals do I need to sample per population?” Until now, researchers have answered this question by calculating the number of individuals needed to detect all of the alleles present in each population using rarefaction analysis, e.g. Kalinowski [1]. However, for population-based studies, detecting all of the alleles present is not as important as ensuring that the frequencies of the alleles detected are representative of those in the total population, and this can be achieved without sampling alleles which are present at very low frequencies. Rare alleles (i.e. those found at frequencies <0.05) are a common feature of microsatellite loci, but very rare alleles (i.e. those found at frequencies <0.01) provide almost no useful information for most population-based analyses even if shared among populations, as their presence may be due to recurrent mutations rather than historical association or contemporary gene flow [2]. Thus, the most informative alleles for assessing genetic structure among populations are those which are common enough to be shared among some individuals, but rare enough to be absent in many individuals. Very rare alleles are useful for some applications, (e.g. parentage analysis or relatedness calculations), but contribute very little information when assessing patterns of genetic diversity or population structure.

Rather than asking how many individuals are needed to detect all alleles present, we argue that a more useful strategy for population genetic sampling design is to consider how many individuals are needed to contain all informative alleles at frequencies that are representative of the population allele frequencies. Given that many population genetic analyses are allele frequency based (e.g. tests of deviation from Hardy-Weinberg, estimates of genetic diversity, AMOVA), or based on comparisons of individual genotypes to ‘population’ allele frequencies (e.g. genotype assignment tests), it is important that the sampled allele frequencies are representative of the true population allele frequencies. Increasing sample size will always increase the accuracy of the allele frequency estimate, but the rate of increase will not be linear. The rate at which accuracy increases should level out as sampling effort increases, while the cost of genotyping more individuals does not. Therefore, the question researchers are really asking is: “at which sample size is the increase in accuracy of allele frequencies too small to warrant the extra cost of sampling more individuals?”

Because we are dealing with simple statistical sampling issue, the sample size required to ensure the sample accurately reflects the allele frequencies of the underlying population should be fairly consistent across populations and taxa. To test this hypothesis, we assessed the impact of sample size on the accuracy of allele frequencies, as well as genetic diversity at each locus and genetic composition across loci (both of which are calculated from sampled allele frequencies), by randomly subsampling four empirical microsatellite genotype datasets representing four ‘populations’ of individuals. These include two large ‘populations’ with over 500 sampled individuals (hairy wood ant, *Formica lugubris*, and black-browed albatross, *Thalassarche melanophris*) and two small ‘populations’ of approximately 100 individuals (British red squirrel, *Sciurus vulgaris*, and black stilts or kakī, *Himantopus novaezelandia*), and span a wide taxanomic range (invertebrates, birds and mammals). The datasets represent a typical range of polymorphism for microsatellite loci used in population studies (two to 13 alleles per locus; H_E_ from 0.267 to 0.807). If the impact of sample size on the accuracy and precision of allele frequencies is similar across all our datasets, then the results presented here should be widely applicable to other studies, and can be used to make sampling design decisions for future population genetic studies based on microsatellite allele frequencies across a range of taxa, population sizes and levels of polymorphism.

This paper does *not* attempt to address the question of how many individuals need to be sampled to detect *all* alleles present in the population. That depends on the number and frequency of alleles in the population and can be addressed on a case by case basis with rarefaction analysis (e.g. using HP-RARE [1]), or regression analysis [3]. This paper also does *not* attempt to address the question of how many individuals need to be sampled to detect genetic differentiation among populations (i.e. assessing population structure). That depends on the level of genetic differentiation that actually exists among the populations as well as variation within populations, and can be assessed with power analysis (e.g. using POWSIM [4], [5]). The aim of this paper is to address the question of how many individuals need to be sampled to provide an accurate estimate of the allele frequencies at each locus and, therefore, expected heterozygosity, within a population. Previous attempts to address this issue have been restricted to *ad hoc* analysis of single taxa datasets [6], [7], [8]. Here we use multiple datasets across a wide variety of taxa to provide generally applicable answers to the following questions: how many individuals per population are needed to 1) detect all the alleles actually present in the population at a frequency ≥0.05; 2) obtain representative allele frequencies; 3) accurately reflect the level of expected heterozygosity (i.e. genetic diversity) and 4) ensure the sample accurately reflects the overall genetic composition (across loci) of the real population? Questions 2 and 3 are closely related, in that accurate allele frequencies will translate into an accurate level of expected heterozygosity, but by looking at the two separately we can assess the impacts of sample size on relatively rare alleles, which have little effect on heterozygosity. Ultimately, if the genetic composition of a population has been accurately characterised, then any downstream population genetic analyses that rely on allele or genotype frequencies should also produce meaningful results.

## Methods

### Random subsampling of empirical datasets

For each of the four species we constructed simulated datasets consisting of 100 replicates each of the following sample sizes: 5, 10, 15, 20, 25, 30, 35, 40, 45, 50, 75 and 100 individuals. Each replicate contained a random subset of individuals from the empirical dataset and replicates were created using a macro in excel, designed to assign each individual in the empirical dataset a random number (between 1 and 10,000), sort the dataset by the random numbers, then select the first 5 (or 10, 15, etc. depending on the sample size category) to a new worksheet, 100 times, resulting in 100 simulated ‘populations’ that are independent, random subsamples of the empirical dataset, at each sample size. Sampling was done without replacement, so no individual was present more than once in the same replicate (as in a real population genetic dataset), but as replicates were independent of each other, the same individual could be present in more than one replicate of the simulated dataset at each sample size. GenAlEx 6.2 [9] was then used to calculate allele frequencies, heterozygosity expected under Hardy-Weinberg Equilibrium (H_E_) and pairwise F_ST_ between the simulated and empirical datasets for each replicate at each sample size. When we refer to the ‘empirical dataset’ we mean the real dataset of 547 ant, 107 squirrel, 616 albatross or 98 kakī individuals (see below for dataset details). Because the kakī dataset comprised fewer than 100 individuals, the largest sample size assessed for this species was 75 individuals. Throughout this paper, ‘individuals’ means diploid individuals.

### Allele detection and frequencies

In order to determine how many individuals need to be sampled to detect all of the ‘informative’ alleles, we first need to decide how common an allele has to be before we consider it likely to be informative. Traditionally, a locus has been considered polymorphic if the most common allele has a frequency of ≤0.95 [2], and therefore the less common allele (at a locus with two alleles) has a frequency of ≥0.05. Given that we need to choose an arbitrary cut-off point, we have considered alleles present at a frequency of ≥0.05 to be ‘informative’. The probability of detecting an allele that occurs at a frequency of 0.05 in the empirical dataset in each simulated dataset can be calculated by taking one minus the probability of non-detection, which is 1−(0.95)^n^ where n equals the number of chromosomes in the sample. From this, we can determine that the sample size needed for at least a 95% probability of detecting an allele that occurs at a frequency of 0.05 is 30 diploid individuals.

To determine the sample size at which every allele at a locus present in the empirical datasets at a frequency of ≥0.05 was detected in at least 95% of the replicates at each sample size, we simply counted the number of replicates for each sample size in which all of the alleles present in the empirical dataset at a frequency of ≥0.05 were detected. To assess the accuracy of the allele frequencies in the simulated datasets, we calculated a) the mean difference between the allele frequency in the simulated and empirical datasets (averaged over the 100 replicates) for each allele, including those with a real frequency <0.05, at each sample size, and 2) the range of differences in allele frequencies for simulated and empirical datasets, for each allele. Given that in a real population study each population is sampled only once, we were not only interested in how inaccurate the sample frequency is likely to be, on average, at a particular sample size, but also what the range of error was likely to be (i.e. how inaccurate a single sample *could* be). For the assessment of mean difference between the simulated and empirical dataset allele frequencies, alleles were grouped into those with a real frequency ≥0.05, between 0.05 and 0.01, and <0.01 to see if sample size had a varying impact with the frequency of the allele in the empirical dataset.

### Expected heterozygosity

Expected heterozygosity (H_E_) is often used to describe levels of genetic diversity as it depends solely on the number and relative frequencies of alleles, and so is a measure of the ‘evenness’ of allele frequencies. As such, for a dataset to accurately reflect levels of genetic diversity, it needs to accurately reflect the level of heterozygosity expected under Hardy-Weinberg Equilibrium (H_E_) in the real population. We used the replicates at each sample size described above to assess the impact of sample size on expected heterozygosity, by calculating the expected heterozygosity for each of the 100 replicates at each sample size, for each locus in each dataset, using GenAlEx [9], and plotted the means (± one standard deviation) and range against sample size for each locus. Due to space restrictions, we have only shown two loci per dataset, one with a high expected heterozygosity in each empirical dataset (i.e. high diversity) and one with low expected heterozygosity, although all loci were examined and showed similar patterns. We have also calculated the mean expected heterozygosity (averaged across loci) for each sample size in each dataset, to demonstrate the impact of sample size on overall H_E_ in typical population genetic studies.

### Genetic composition

With microsatellite data we usually genotype each individual at many loci and it's the genetic composition across all those loci that is used to answer questions about gene flow, differentiation, origins of individuals, etc. Therefore, we need to be sure that the sampled genotypes accurately reflect the genetic composition of the real population both within and across loci. One way to test this is to see how sample size affects genetic distance between the simulated and the empirical datasets. When genetic distance is very small, all replicates are similar to the empirical dataset, so we know that sampling that number of individuals should give consistent and accurate results. We have calculated the pairwise genetic distance between the simulated and the empirical datasets (as pairwise F_ST_) for each of the 100 replicates at each sample size for each of the four datasets using GenAlEx [9], and plotted the mean and standard deviations against sample size. We also calculated Nei's genetic distance between the simulated and empirical datasets at each sample size (data not shown), but obtained an almost identical pattern to the pairwise F_ST_ values, so we have only presented pairwise F_ST_ data.

### Empirical datasets

The ant dataset consisted of microsatellite genotypes at nine loci (FL12, FL20, FL21 and FL29, [10]; FE13, FE16, FE17, FE37 and FE38, [9]) for 547 hairy wood ant (*Formica lugubris*) diploid female individuals, collected over a small area (approximately 30 m×100 m) in Slaley Forest, England, in 2004 (see Supporting Information S1 for genotyping details). The ants were collected from 30 different nests for a population structure study, and do show some differentiation among nests over that range (F_ST_ = 0.07; unpublished data), but for the purpose of this study individuals have been pooled as a single ‘population’. This species is not strictly eusocial, but rather there are many reproductive individuals per nest, and reasonably high within nest genetic variation [11]. The population contained a total of two to 13 alleles per locus and heterozygosities expected under Hardy-Weinberg Equilibrium (H_E_) varied from 0.267 to 0.779, with an average H_E_ of 0.556 (Table S1, Supporting Information S1). The inclusion of datasets containing some population structure (i.e. individuals from genetically different populations) is unlikely to have any impact upon the comparison of empirical and simulated datasets, as the structure should exist in both the empirical and simulated datasets to the same extent (at least in the larger samples). At smaller sample sizes the true structure may not be accurately represented due to sampling error, but that is what this study is assessing: how accurately do the samples represent the true population allele frequencies (whether structured or not) at each sample size?

The squirrel dataset consisted of microsatellite genotypes at five loci (Scv3, Scv8, Scv9, Scv10 and Scv23, [12]) for 107 British red squirrel (*Sciurus vulgaris*) individuals collected across northern England & southern Scotland (11 populations) over the last 100 years (samples were obtained from dried museum skins). The genotypes were collected as part of a published population genetic study and populations were moderately differentiated across space and time (F_ST_ = 0.16, [13]), but again have been pooled as a single ‘population’ for the purpose of this study. There were between three and 10 alleles per locus and H_E_ ranged from 0.385 to 0.775, with an average H_E_ of 0.579 (Table S1).

The albatross dataset consisted of microsatellite genotypes at seven loci (D22, De11, D5, D27, D9, D21 and De35) for 616 adult black-browed albatross (*Thalassarche melanophris*) individuals collected from a single population (Bird Island, South Georgia), as part of a published population genetic study [14], [15]. There were between six and 13 alleles per locus and H_E_ ranged from 0.268 to 0.807, with an average H_E_ of 0.591 (Table S1).

The kakī dataset consisted of microsatellite genotypes at eight loci (Kakī_2, Kakī_9, Kakī_12, Kakī_13, Kakī_21, Kakī_27, Kakī_40 and Kakī_di7, [16]) for 98 kakī (*Himantopus novaezelandiae*) individuals collected as part of a published study assessing hybridisation and introgression [17]. There were between two and five alleles per locus and expected heterozygosity (H_E_) varied from 0.291 to 0.712, with an average H_E_ of 0.531 (Table S1). The kakī dataset represents 96% of all individuals of this critically endangered species known to be alive in 2007.

The albatross and kakī datasets included individuals with missing data at no more than one locus (albatross: 2% of individuals missing one locus; kakī:11% of individuals missing one locus), while the ant and squirrel datasets did not contain any missing data.

## Results

### Question 1: How many individuals are needed for a 95% probability to detect alleles present in the empirical dataset at a frequency of ≥0.05?

**Figure 1:**
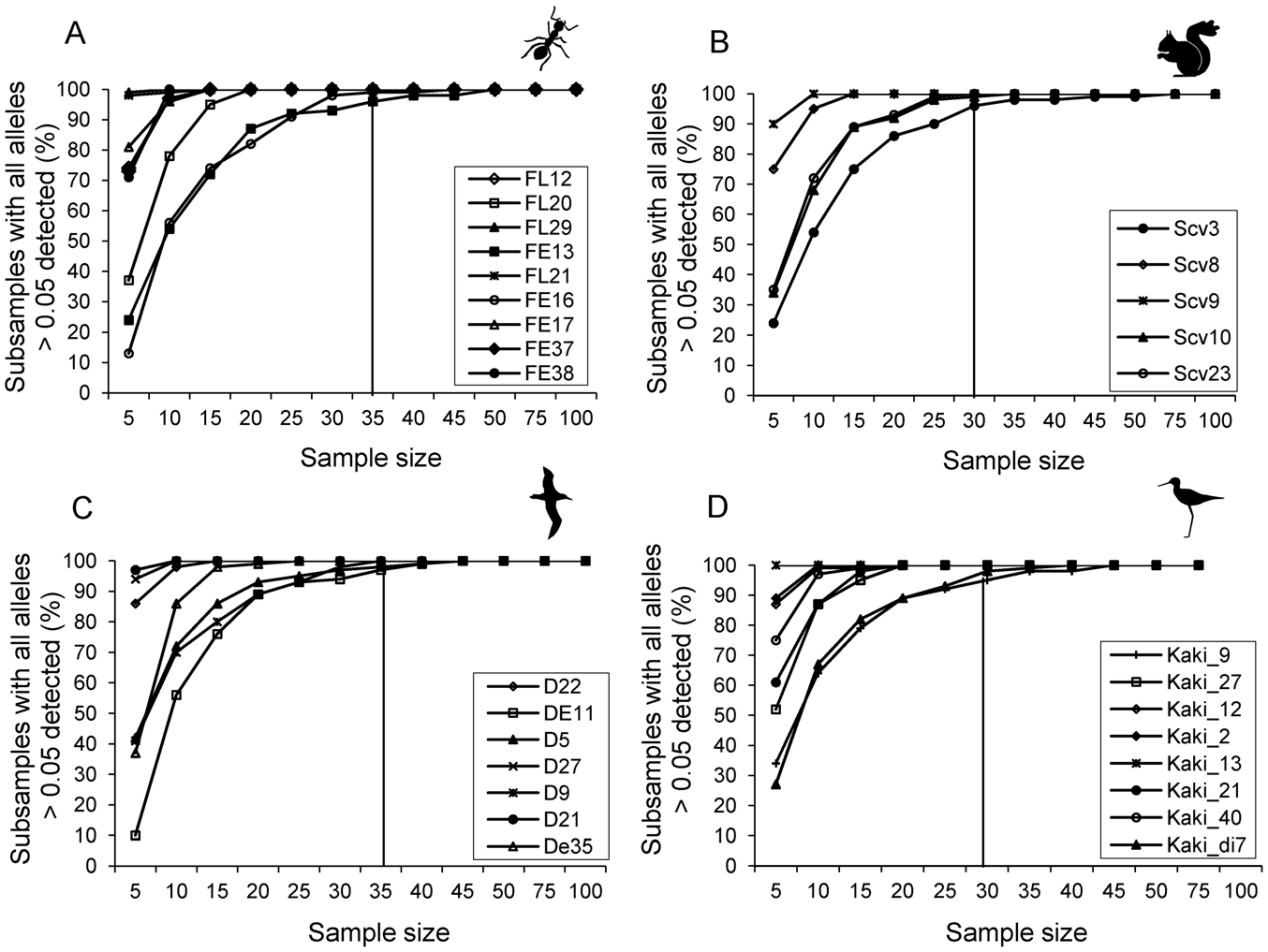
Impact of sample size on allele detection. Percentage of samples in which all alleles at a real frequency ≥0.05 at each locus were detected at each sample size (of the 100 random replicates per size), for A) the ant dataset, B) the squirrel dataset, C) the albatross dataset and D) the kakī dataset. The vertical line shows the sample size at which all alleles at a real frequency ≥0.05 at all loci were detected in ≥95% of replicates.

The sample size at which all alleles present in the empirical dataset at a frequency ≥0.05 were detected in at least 95% of the replicates varied by locus, and ranged between a sample size of five and 35 in the ant dataset, between 10 and 30 in the squirrel dataset, between five and 35 in the albatross dataset, and between five and 30 in the kakī dataset (Figure 1). Loci with equally frequent alleles (e.g. ant locus FE38) required a much smaller sample size (n = 5) to detect all of the alleles present in the empirical dataset at a frequency ≥0.05, than loci with one or more alleles at a frequency just above 0.05 (e.g. ant locus FE16, required n = 35). These results support the sample size of 30 theoretically required for a 95% probability of detecting an allele at a frequency of 0.05.

### Question 2: How many individuals are needed to get representative allele frequencies?

**Figure 2:**
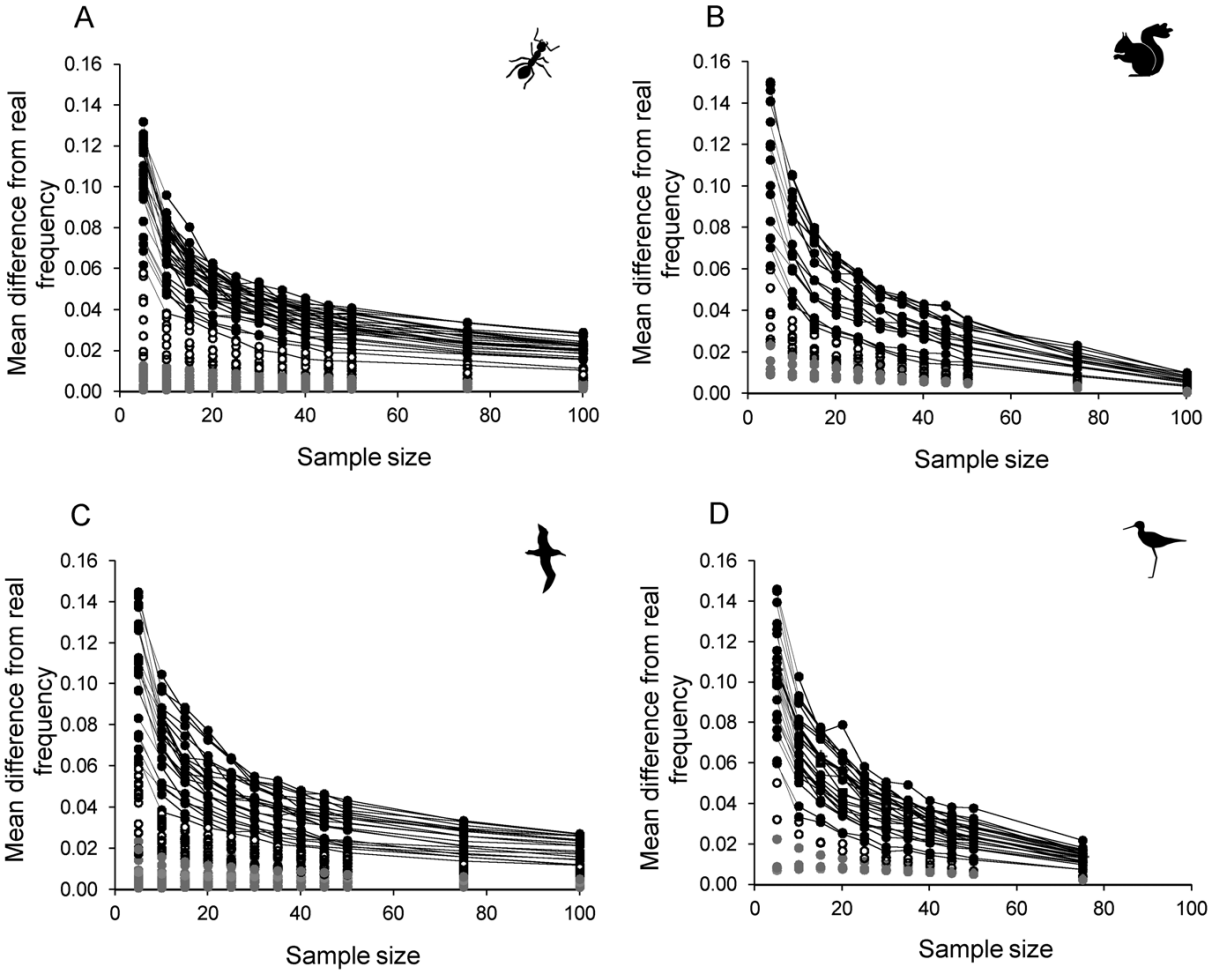
Impact of allele frequency and sample size on the accuracy of mean sample allele frequency. Mean difference from the real allele frequency for each sample size (of the 100 random replicates per size) for A) the ant dataset, B) the squirrel dataset, C) the albatross dataset and D) the kakī dataset. Black circles represent alleles with a real frequency ≥0.05 (data for the same allele at different sample sizes linked by a line), white circles represent alleles with a real frequency between 0.05 and 0.01, and grey circles represent alleles with a real frequency ≤0.01.

Increasing sample size clearly results in a decrease in the mean difference in allele frequencies between the simulated and empirical datasets for each allele (Figure 2). However, that incremental improvement in accuracy decreases with increasing sample size, so what we really want to know is at which point is the increase in accuracy obtained by additional sampling outweighed by the extra cost of more sampling and genotyping? That point will change depending on the costs of sampling for each study (some species are considerably cheaper to sample than others), but our data suggest that the gain in accuracy is small beyond approximately 25 to 30 individuals, particularly when sampling from relatively large populations (Figure 2A,C). When the population being sampled is small, the incremental increase in accuracy of the allele frequency with increasing sample size may be greater, as seen with the steeper decrease in mean difference in allele frequencies for the squirrel (Figure 2B) and kakī (Figure 2D) datasets, as the incremental increase of five individuals represents a relatively larger proportion of the population being sampled. However, even with 100 of the 107 squirrel individuals sampled, the allele frequencies still differ slightly (approx. 2%) from those of the empirical dataset.

Despite the differences in taxa, level of genetic variation, level of population structure within the empirical dataset, and the size of population being sampled, all four datasets showed similar patterns in allele frequencies between the simulated and empirical datasets at each sample size (Figure 2), with the degree of difference associated with the real allele frequency. For example, the range of mean difference in allele frequency for alleles present in the empirical dataset at a frequency ≥0.05 (Figure 2, black circles) at a sample size of five was almost identical across the four datasets: 0.061 to 0.132 (ants); 0.062 to 0.150 (squirrels); 0.061 to 0.145 (albatross); and 0.060 to 0.146 (kakī). At each sample size, the mean difference in allele frequency was strongly correlated with the frequency of that allele in the empirical dataset, with correlation coefficients ranging from r = 0.627 (sample size 75) to r = 0.774 (sample size 5; n = 170 alleles pooled across all four datasets, P <0.0001 for all correlation coefficients). The greatest difference between allele frequencies in the two datasets were for alleles with a frequency close to 0.5 in the empirical dataset, where the mean frequency difference decreased as the allele frequency in the empirical dataset moved towards zero or one. The mean difference between replicate and real frequencies for rare alleles (frequency below 0.01) was almost unaffected by sample size (Figure 2, grey circles).

**Figure 3:**
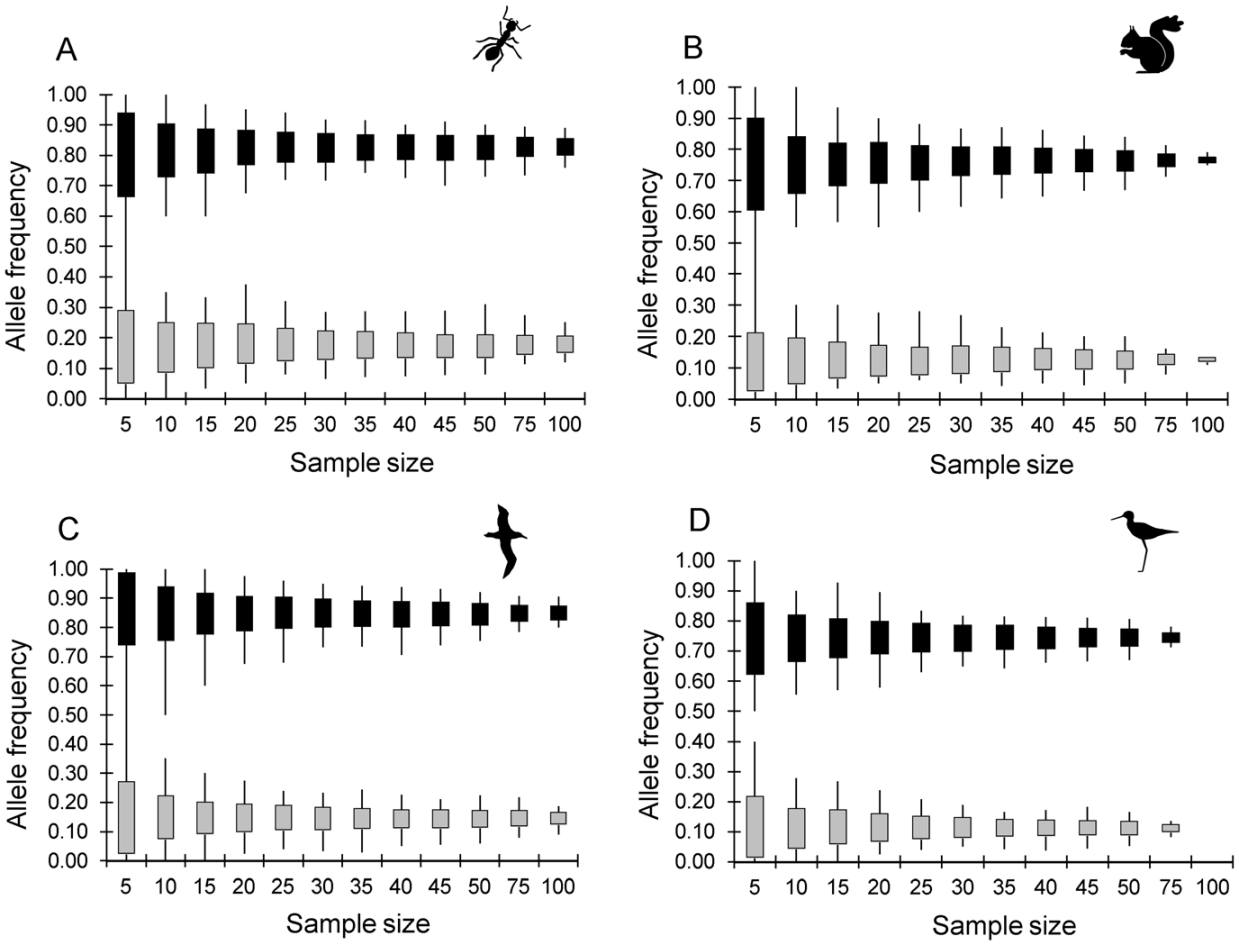
Impact of sample size on the precision of sample allele frequencies. The range (lines) and mean ± one standard deviation (solid boxes) of allele frequencies of the 100 random replicates at each sample size for one common and one relatively rare allele in A) the ant dataset, B) the squirrel dataset, C) the albatross dataset and D) the kakī dataset. The alleles are: A) allele 159 at locus FE16 (grey boxes, real frequency  = 0.176) and allele 116 at locus FE17 (black boxes, real frequency  = 0.833); B) allele 196 at locus Scv8 (grey boxes, real frequency  = 0.126) and allele 162 at locus Scv23 (black boxes, real frequency  = 0.766); C) allele 187 at locus De35 (grey boxes, real frequency  = 0.147) and allele 165 at locus D5 (black boxes, real frequency  = 0.852); and D) allele 241 at locus Kakī_21 (grey boxes, real frequency  = 0.112) and allele 200 at locus Kakī_27 (black boxes, real frequency  = 0.745).

The range of allele frequencies obtained among each of the 100 replicates at each sample size was similar among alleles, among loci and among datasets (Figure 3). Due to space restrictions, we have only presented the data for one high frequency allele and one low frequency allele (from different loci) per dataset, but all alleles gave similar patterns. For the ant and albatross datasets there appears to be little decrease in either range or standard deviation of the sample allele frequencies with increasing sample size beyond approximately 25 individuals (Figure 3A,C), suggesting that there is not much to be gained from sampling more than 25 individuals per population when the population size is large. The kakī dataset also shows little improvement in accuracy of allele frequencies when sample size increases above 25 individuals, apart from at a sample size of 75, where the majority of individuals in the population have been sampled (Figure 3D). For the squirrel dataset, there is little improvement in the accuracy of allele frequencies beyond about 30 individuals, until the sample size reaches 100, where almost all individuals in the population have been sampled (Figure 3B).

### Question 3: How many individuals are needed to accurately reflect the real level of expected heterozygosity (i.e. genetic diversity) in the population?

**Figure 4:**
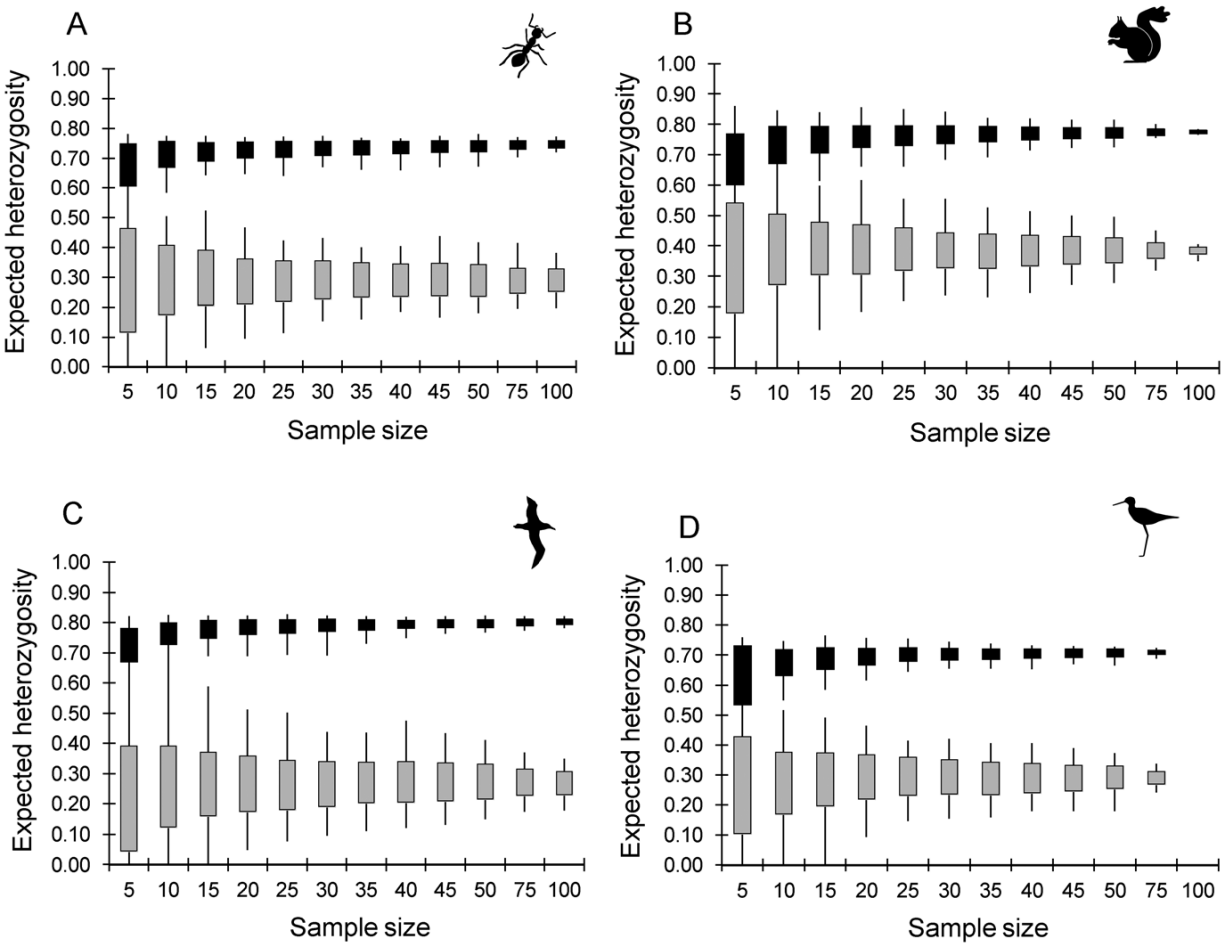
Impact of sample size on the accuracy and precision of sample heterozygosity. The range (lines) and mean ± one standard deviation (solid boxes) of expected heterozygosity (H_E_) for the 100 random replicates at each sample size for a locus with high and a locus with low expected heterozygosity in the empirical dataset for A) the ant dataset, B) the squirrel dataset, C) the albatross dataset and D) the kakī dataset. Loci are: A) locus FE37 (real H_E_ = 0.75, black boxes) and locus FE17 (real H_E_ = 0.29, grey boxes); B) locus Scv3 (real H_E_ = 0.78, black boxes) and locus Scv23 (real H_E_ = 0.39, grey boxes); C) locus De11 (real H_E_ = 0.807, black boxes) and locus D5 (real H_E_ = 0.268, grey boxes); and D) locus Kakī_21 (real H_E_ = 0.712, black boxes) and locus Kakī_40 (real H_E_ = 0.291, grey boxes).

**Figure 5:**
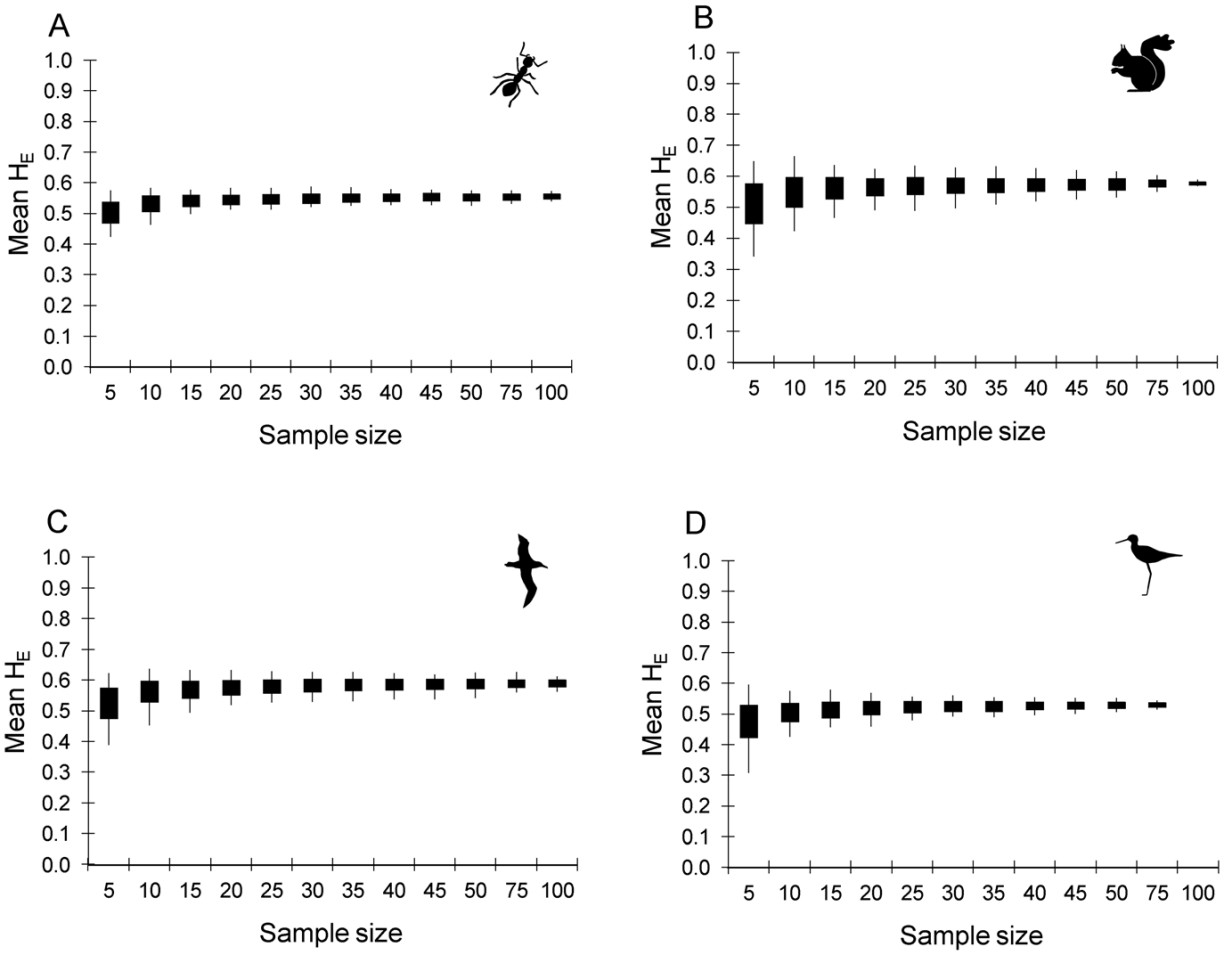
Impact of sample size on the accuracy and precision of mean heterozygosity across loci. The range (lines) and mean ± one standard deviation (solid boxes) of mean expected heterozygosity (H_E_) for the 100 random replicates at each sample size for A) the ant dataset (9 loci), B) the squirrel dataset (5 loci), C) the albatross dataset (7 loci) and D) the kakī dataset (8 loci).

The expected heterozygosities of the replicates were much more precise (lower range and standard deviation at each sample size) for loci with high H_E_ than those with low H_E_ (Figure 4, 5). The accuracy and precision of H_E_ did not increase greatly beyond a sample size of 15 (ants & albatross, Figure 4A,C) to 20 (squirrels & kakī, Figure 4B,D) for the loci with highest heterozygosity in each dataset. The range of H_E_ values for loci with low real H_E_ was considerably greater than loci with high H_E_ in all datasets, and H_E_ was quite variable for loci with low polymorphism in the simulated datasets, even with a sample size as high as 100. This suggests that increasing sample size beyond 20 individuals will have little impact on the accuracy of H_E_ either for highly polymorphic loci, because a sample size of 20 will provide an accurate estimate of H_E_, or for loci with low polymorphism, because the estimate of H_E_ will be inaccurate unless almost all of the individuals in the population are sampled. The accuracy of H_E_ also increased with the ‘evenness’ of allele frequencies. For example, in the ant dataset loci FE38 and FE16 have similar real H_E_ (0.75 and 0.78 respectively), but all four alleles at locus FE38 have similar frequencies (0.209 to 0.276), while FE16 has 13 alleles ranging from a frequency of 0.001 to 0.316. At a sample size of 25, the mean H_E_ at both loci (FE38 and FE16) was very close to the real value (0.018 and 0.016 below the real H_E_ respectively), but the range of H_E_ values among the 100 replicates was much lower for FE38 (0.678–0.749) than for FE16 (0.669–0.834). Thus a single sampling event is likely to result in a more accurate H_E_ at loci with equally frequent alleles.

Both the accuracy and precision of mean H_E_ increased with increasing sample size, but much of the increase occurs when sample size is increased from 5 to 20 individuals (Figure 5). Increasing sample size above 20 individuals appears to have little impact on the mean H_E_ or its standard deviation in all four datasets. We can also see the impact on H_E_ of increasing the number of loci sampled, with the dataset with the smallest number of loci (squirrels, 5 loci) having less precision than the dataset with the largest number of loci (ants, 9 loci, Figure 5).

### Question 4: How many individuals are needed to ensure the sample accurately reflects the overall genetic composition of the empirical dataset?

**Figure 6:**
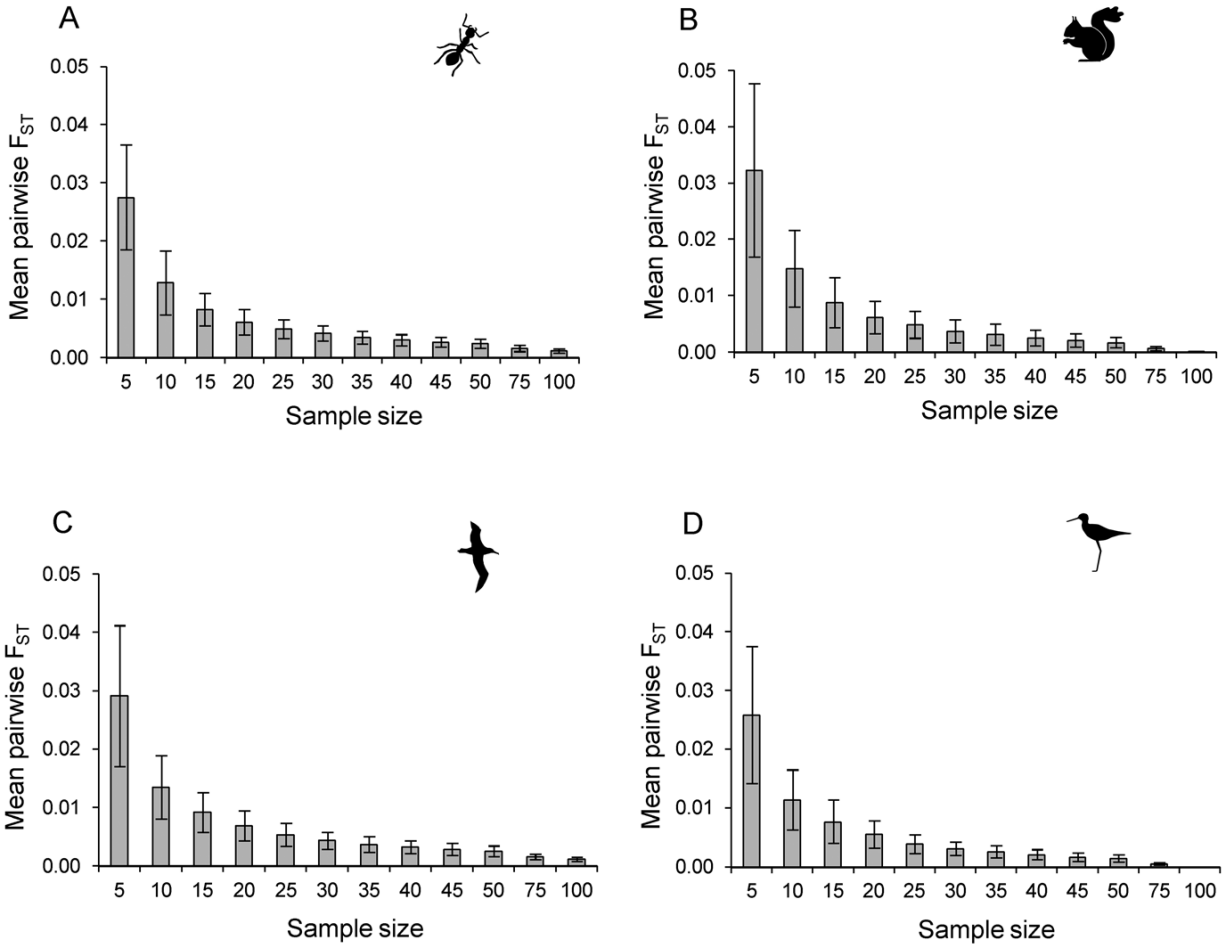
Impact of sample size on mean genetic distance between samples and the true population. Mean pairwise F_ST_ between the 100 random replicates and the empirical dataset for A) the ant dataset, B) the squirrel dataset, C) the albatross dataset and D) the kakī dataset at each sample size. Error bars are standard deviation.

As with the allelic diversity, we expected that the genetic distance among simulated and the empirical datasets would decrease as the sample size increased, but this decrease would not be linear. Therefore, we are looking for the point at which the increase in accuracy gained by adding extra samples (non-linear) is outweighed by the increase in cost (generally linear) of obtaining those additional samples. From figure 6, we can see that the incremental decrease in pairwise F_ST_ is small beyond a sample size of approximately 25 for all four datasets, until almost all individuals are sampled (sample size of 100 and 75 for squirrels and kakī respectively; Figure 6B,D). We tested whether the pairwise F_ST_ values (between each replicate and the empirical dataset) were significantly different among sample sizes using ANOVA, after arcsine squareroot transformation of the F_ST_ values to approximate a normal distribution (using Statistica 8.0). In all datasets, pairwise F_ST_ was highly significantly different across the range of sample sizes (ants: F_11,1188_ = 711.39, P <0.0001; squirrels: F_11,1188_ = 511.80, P <0.0001; albatross: F_11,1188_ = 569.69, P <0.0001; kakī: F_10,1089_ = 538.42, P <0.0001), but the difference was primarily among samples of size 5 to 20. From a sample size of 25 upwards, there was no significant difference in pairwise F_ST_ from one sample size to the next for the ant, albatross and kakī datasets, apart from the increment from the disproportionate jump from 50 to 75 individuals (Tukey HSD posthoc comparisons). In the squirrel dataset, the incremental decreases in pairwise F_ST_ were not significant from one sample size to the next, between sample sizes of 20 to 50, but with significant reduction in pairwise F_ST_ from 50 to 75 and 75 to 100 individuals (Tukey HSD posthoc comparisons) where the increases in sample size were larger.

## Discussion

Sample size had a consistent effect on the accuracy of allele frequencies, expected heterozygosity and genetic composition across datasets. The mean difference in allele frequency between the simulated datasets and empirical dataset was almost identical when compared across the four taxa at each sample size, except for samples where almost all the individuals in the population were sampled (75 for kakī and 100 for squirrels, Figure 2). The relationship between sample size and mean genetic distance between replicates and the empirical dataset was also similar among the four taxa, as were levels of variance among replicate allele frequencies for all alleles, irrespective of the locus or taxon. This suggests that the results obtained here will be widely applicable to other studies, and that the pattern of difference in allele frequencies, heterozygosities and genetic composition across sample sizes seen here can be used to make sampling design decisions for future microsatellite-based population genetic studies. The ‘optimal’ sample size, i.e. the size beyond which the increase in accuracy or precision is very small, varies for each measure we have assessed (detection of alleles, allele frequencies, expected heterozygosity and genetic composition), so we have presented recommendations for each measure separately (below), as well as an overall recommendation for sampling for microsatellite-based population genetic studies incorporating most or all of these measures.

The accuracy of replicate allele frequencies was dependent, to some extent, on the frequency of that allele in the empirical dataset. The mean difference in allele frequency (from simulated compared to the empirical dataset) was greatest for alleles with a real frequency close to 0.5 at any sample size, and the difference decreased as the allele frequency moved towards zero or one. The accuracy of sampling frequencies of rare alleles (frequency between 0.01 and 0.05) was only slightly affected by sample size, with the mean difference in allele frequency being slightly greater at smaller sample sizes than large, while the accuracy of sampling frequencies of very rare alleles (real frequency <0.01) appeared to be unaffected by sample size. This is not surprising as very rare alleles are unlikely to be sampled, and therefore, the difference in allele frequency will be equal to the actual allele frequency, which is a small number. When rare alleles are sampled, the sampled allele frequency is likely to be overestimated, especially at small sample sizes. This adds to our argument that sampling design should not be driven by the need to sample all the rare alleles present in a population, since they not only add very little information to population-based studies, but our results suggest that on average the accuracy of frequencies of rare alleles does not improve substantially with increasing sample size.

Although the mean difference in allele frequency was higher for alleles close to a frequency of 0.5, than those nearer zero or one at each sample size, the impact of sample size on the precision of sampled allele frequencies was similar across all alleles with a real frequency of at least 0.05. In all cases, the range and standard deviation of sampled allele frequencies decreased with increasing sample size, but the incremental increase in precision (decrease in range and standard deviation) was less as sample size increased. Therefore, there is a point beyond which increased sampling will have little impact on the accuracy and precision of estimates of allele frequency, and, in our opinion, that point is reached at approximately 25 to 30 individuals. The replicates at each sample size demonstrated that all alleles with a frequency ≥0.05 in the empirical dataset were detected in ≥95% of replicates when the sample size was between 30 and 35, which is in agreement with the theoretical calculation of 30 diploid individuals required for a 95% probability of detecting an allele at a frequency of 0.05. We therefore recommend a sample size of approximately 25 to 30 individuals per population to ensure most of the informative alleles are sampled at frequencies that reflect those in the total population.

The accuracy and precision of expected heterozygosities appeared to be affected by the level of polymorphism at each locus. The sample size required for an expected heterozygosity that was representative of the real H_E_ was lower for loci with high expected heterozygosity in the empirical dataset compared to those with low real expected heterozygosities. This is in contrast to previous suggestions that large sample sizes are needed to accurately describe population structure at highly variable loci [18], [19], [20], but is consistent with recent work assessing the impact of sample size on genetic differentiation [21]. From our datasets, it appears that H_E_ of loci with high polymorphism (e.g. H_E_ above 0.7; common among microsatellite loci) can be accurately estimated from small samples, only 15 to 20 individuals, while the sampled H_E_ of loci with low polymorphism (e.g. H_E_ below 0.3) can be considerably different from the real population H_E_ even with a sample size of 100. For example, at ant locus FE17, the replicate H_E_ varied from 0.197 to 0.381 at a sample size of 100 (real H_E_ = 0.290) while locus FE37 replicate H_E_ only varied from 0.720 to 0.773 (real H_E_ = 0.750) at that same sample size. The precision of the H_E_ estimate for locus FE17 at sample size of 100 (standard deviation of 0.038) was actually lower than the precision of the H_E_ estimate for FE37 at a sample size of only 15 (standard deviation  = 0.031). This suggests that estimates of H_E_ based on loci with low polymorphism are likely to be less accurate than those based on highly polymorphic loci, even when reasonably large samples are taken from each population. One of the implications of this lower accuracy of H_E_ estimates for loci with low polymorphism is that any analyses that utilise deviation from Hardy-Weinberg Equilibrium to assess process (e.g. assessment of inbreeding from F_IS_) or methodology (e.g. deficit of heterozygotes to assess null alleles) will be much less accurate when based on loci with low polymorphism compared to loci with high polymorphism. The accuracy and precision of mean H_E_ (across loci) increased with increasing sample size from 5 to 20 individuals, but increasing sample size beyond 20 individuals appeared to have little impact on the precision or accuracy of mean H_E_. Thus in a typical microsatellite based population study that includes a mixture of loci with high and low polymorphism, generally mean H_E_ can be accurately determined from samples as small as 20 individuals.

The genetic composition of samples was more similar to the underlying population as sample size increased (measured as pairwise F_ST_), but as with allele frequency estimates, the incremental increase in accuracy (i.e. decrease in the genetic distances) decreased with increasing sample size. The incremental decrease in pairwise F_ST_ with increasing sample size (addition of five individuals per increment) was not significant beyond a sample size of 20 to 25, in all four datasets.

Small samples are clearly subject to large errors when estimating allele frequencies and expected heterozygosity. For example, in all four datasets, frequencies of any common allele among replicate samples of 10 individuals were up to 0.4 different from each other (see range of high frequency alleles, Figure 3) and, averaged across replicates, were up to 0.1 different from the real population allele frequency (Figure 2). The level of error is approximately half of this at a sample size of 30 in all four datasets. Mean expected heterozygosity was also much less precise at a sample size of 10 compared to a sample size of 30. The range of mean H_E_ across replicates at sample size of 10 was 0.12 (ants) up to 0.24 (squirrels); while at a sample size of 30 the range of mean H_E_ across replicates was 0.05 (ants and kakī) to 0.10 (albatross) (see range, Figure 5). Therefore, population genetic studies based on very small samples need to recognise the potentially very large error in small sample allele frequencies, and small samples (under 20 individuals) should be avoided if possible. This does not apply to situations where a sample is small because the population being sampled is very small (i.e. almost all individuals are sampled). It is clear from Figures 3, 4 and 5 that when the sample size is close to population size (e.g. squirrels at a sample of 100 out of a population of 107, and kakī at a sample of 75 out of 98), the accuracy and precision of both allele frequencies and expected heterozygosity is very high. Note that when small samples are unavoidable, our results suggest that the accuracy of mean expected heterozygosity can be increased by increasing the number of loci genotyped.

The data presented here clearly demonstrate the relationship between sample size and the accuracy of allele frequencies, expected heterozygosity and genetic composition and show the level of error to expect at each sample size for microsatellite loci across a wide range of polymorphism. In our opinion, the increase in accuracy of samples with increasing sample size is not likely to warrant the extra data collection cost above a sample size of 25 to 30 diploid individuals for microsatellite-based population genetic studies incorporating analyses using allele frequency and heterozygosity information. Given that we found a consistent relationship between sample size and accuracy across four diverse datasets, we are confident the data presented here can be used by researchers around the globe to assess expected levels of error when considering sampling design for population genetic studies in a myriad of taxa.

## Supporting Information

Supporting Information S1 (DOCX)

Table S1 **Allele frequencies at each locus for each dataset.** Ants (*Formica lugubris*, *n* = 547); squirrels (*Sciurus vulgaris*, *n* = 107), albatross (*Thalassarche melanophris*, *n* = 616), and kakī (*Himantopus novaezelandiae*, *n* = 98). Both the albatross and kakī dataset contained missing data for some loci, number of individuals genotyped at each locus is indicated below locus name. There were no missing data in the ant and squirrel datasets.
(DOC)
