# Supplementary material for: Sampling for Microsatellite-Based Population Genetic Studies: 25 to 30 Individuals per Population Is Enough to Accurately Estimate Allele Frequencies
Source: PLoS One. 2012 Sep 12;7(9):e45170. doi: 10.1371/journal.pone.0045170 (PMC3440332; doi:10.1371/journal.pone.0045170)
Supplement: Supporting Information S1 — (DOCX) [file pone.0045170.s001.docx]

**Supporting information**

**Collection of microsatellite genotypes for the ant (formica lugubris) data set:**

Ant genotypes were obtained as follows: DNA was extracted from whole ants using a CTAB with chloroform method [1]. For each individual, nine microsatellite loci were amplified in six PCR reactions. Loci FL12, FL20, FL21 and FL29 were originally isolated from *F. lugubris* [2] and loci FE13, FE16, FE17, FE37 and FE38 were originally isolated from *F. exsecta* [3]. Loci FL12 and FE13 were multiplexed in 15 µl reactions containing 1x *Taq* buffer (16 mM (NH_4_)_2_SO_4_, 67 mM Tris-HCl, 0.01% Tween-20), 2.5mM MgCl_2_, 0.2mM each dNTP, 0.33μM each primer, and 0.6U *Taq* (Bioline), with 0.5µl template DNA. The PCR reaction cycle was as follows: 95ºC for 12 mins, 10 cycles of 94ºC for 15 sec, 47°C for 15 sec, 72ºC for 15 sec, followed by 30 cycles of 89ºC for 15 sec, 47°C for 15 sec, 72ºC for 15 sec, then a final extension of 72ºC for 10 mins. Loci FE16, FE17 and FE37 were multiplexed in 15 µl reactions containing 1x *Taq* buffer, 2.5mM MgCl_2_, 0.2mM each dNTP, 0.17μM each FE16 primer, 0.26μM each FE17 primer, 0.33μM each FE37 primer, 0.6U *Taq* (Bioline), with 0.5µl template DNA. Reaction cycle was as above. The remaining four loci were amplified singly in 15 µl reactions containing 1x *Taq* buffer (16 mM (NH_4_)_2_SO_4_, 67 mM Tris-HCl, 0.01% Tween-20), 2.5mM MgCl_2_, 0.2mM each dNTP, 0.33μM each primer, 0.6U *Taq* (Bioline), with 0.5µl template DNA, under the same reaction cycle as above, apart from FL21 and FL29 for which the annealing temperature was 50°C. The 5’ end of the forward primer for each locus was fluorescently labelled with either FAM (6-FAM), JOE or TAMRA (ABI dyes). Fluorescently labelled fragments were detected on an ABI 310 genetic analyser, with ROX 500 internal size standard, and analysed and sized using Genescan^®^ software (Applied Biosystems).

**References.**

1. Weising K, Nybom H, Wolff K, Meyer W (1995) DNA fingerprinting in plants and fungi. Boca Raton, USA, CRC Press.
2. Chapuisat M (1996) Characterization of microsatellite loci in *Formica lugubris B* and their variability in other ant species. Mol. Ecol. 5: 599-601.
3. Gyllenstrand N, Gertsch PJ, Pamilo P (2002) Polymorphic microsatellite DNA markers in the ant *Formica exsecta*. Mol. Ecol. Notes 2: 67-69.
