# Supplementary material for: Sampling for Microsatellite-Based Population Genetic Studies: 25 to 30 Individuals per Population Is Enough to Accurately Estimate Allele Frequencies
Source: PLoS One. 2012 Sep 12;7(9):e45170. doi: 10.1371/journal.pone.0045170 (PMC3440332; doi:10.1371/journal.pone.0045170)
Supplement: Table S1 — Allele frequencies at each locus for each dataset. Ants (Formica lugubris, n = 547); squirrels (Sciurus vulgaris, n = 107), albatross (Thalassarche melanophris, n = 616), and kakī (Himantopus novaezelandiae, n = 98). Both the albatross and kakī dataset contained missing data for some loci, number of individuals genotyped at each locus is indicated below locus name. There were no missing data in the ant and squirrel datasets. (DOC) [file pone.0045170.s002.doc]

**Table S1. Allele frequencies at each locus for each dataset.**

| **Ants:** |  |  | **Squirrels:** | |  | | **Albatross:** | |  | |  | **Kakī:** |  | |  | |
| --- | --- | --- | --- | --- | --- | --- | --- | --- | --- | --- | --- | --- | --- | --- | --- | --- |
| **Locus** | **Allele** | **Freq.** | **Locus** | **Allele** | | **Freq.** | **Locus** | **Allele** | | **Freq.** | | **Locus** | | **Allele** | | **Freq.** |
| **FL12** | **102** | 0.592 | **Scv3** | **183** | | 0.023 | **D22** | **105** | | 0.222 | | **KAKĪ_9** | | **119** | | 0.201 |
|  | **106** | 0.188 |  | **187** | | 0.023 | (*n* = 616) | **107** | | 0.433 | | (*n* = 97) | | **127** | | 0.351 |
|  | **108** | 0.045 |  | **203** | | 0.009 |  | **109** | | 0.032 | |  | | **131** | | 0.397 |
|  | **110** | 0.175 |  | **205** | | 0.037 |  | **110** | | 0.296 | |  | | **139** | | 0.052 |
| **FL20** | **109** | 0.144 |  | **207** | | 0.313 |  | **173** | | 0.001 | | **KAKĪ_27** | | **188** | | 0.005 |
|  | **111** | 0.481 |  | **209** | | 0.266 |  | **181** | | 0.001 | | (*n* = 96) | | **192** | | 0.005 |
|  | **113** | 0.006 |  | **211** | | 0.051 |  | **183** | | 0.011 | |  | | **200** | | 0.745 |
|  | **115** | 0.087 |  | **213** | | 0.220 |  | **185** | | 0.002 | |  | | **204** | | 0.141 |
|  | **116** | 0.281 |  | **215** | | 0.047 |  | **193** | | 0.003 | |  | | **208** | | 0.104 |
|  | **117** | 0.001 |  | **217** | | 0.009 | **De11** | **174** | | 0.185 | | **KAKĪ_12** | | **245** | | 0.740 |
|  | **118** | 0.001 | **Scv8** | **196** | | 0.126 | (*n* = 613) | **180** | | 0.165 | | (*n* = 98) | | **249** | | 0.224 |
| **FL29** | **175** | 0.005 |  | **198** | | 0.472 |  | **190** | | 0.215 | |  | | **253** | | 0.036 |
|  | **179** | 0.364 |  | **200** | | 0.402 |  | **192** | | 0.266 | | **KAKĪ_2** | | **132** | | 0.216 |
|  | **181** | 0.579 | **Scv9** | **191** | | 0.014 |  | **194** | | 0.103 | | (*n* = 97) | | **136** | | 0.464 |
|  | **183** | 0.003 |  | **193** | | 0.322 |  | **196** | | 0.066 | |  | | **140** | | 0.320 |
|  | **185** | 0.004 |  | **195** | | 0.650 |  | **198** | | 0.001 | | **KAKĪ_13** | | **175** | | 0.541 |
|  | **191** | 0.045 |  | **197** | | 0.014 | **D5** | **159** | | 0.033 | | (*n* = 98) | | **187** | | 0.459 |
|  | **204** | 0.001 | **Scv10** | **68** | | 0.196 | (*n* = 609) | **161** | | 0.014 | | **KAKĪ_21** | | **229** | | 0.372 |
| **FE13** | **186** | 0.095 |  | **70** | | 0.005 |  | **163** | | 0.056 | | (*n* = 94) | | **233** | | 0.197 |
|  | **189** | 0.056 |  | **72** | | 0.061 |  | **165** | | 0.852 | |  | | **237** | | 0.314 |
|  | **192** | 0.849 |  | **74** | | 0.014 |  | **167** | | 0.039 | |  | | **241** | | 0.112 |
| **FL21** | **212** | 0.718 |  | **76** | | 0.500 |  | **171** | | 0.007 | |  | | **245** | | 0.005 |
|  | **214** | 0.282 |  | **78** | | 0.220 | **D27** | **84** | | 0.002 | | **KAKĪ_40** | | **122** | | 0.010 |
| **FE16** | **158** | 0.277 |  | **82** | | 0.005 | (*n* = 615) | **92** | | 0.001 | | (*n* = 97) | | **132** | | 0.830 |
|  | **159** | 0.176 | **Scv23** | **138** | | 0.009 |  | **94** | | 0.005 | |  | | **140** | | 0.139 |
|  | **160** | 0.053 |  | **162** | | 0.766 |  | **96** | | 0.294 | |  | | **145** | | 0.021 |
|  | **161** | 0.316 |  | **164** | | 0.065 |  | **98** | | 0.665 | | **KAKĪ_di7** | | **190** | | 0.052 |
|  | **162** | 0.096 |  | **166** | | 0.005 |  | **100** | | 0.018 | | (*n* = 96) | | **208** | | 0.135 |
|  | **163** | 0.012 |  | **168** | | 0.154 |  | **102** | | 0.015 | |  | | **210** | | 0.526 |
|  | **164** | 0.001 |  |  | |  | **D9** | **80** | | 0.002 | |  | | **212** | | 0.005 |
|  | **167** | 0.012 |  |  | |  | (*n* = 616) | **82** | | 0.002 | |  | | **214** | | 0.281 |
|  | **168** | 0.002 |  |  | |  |  | **84** | | 0.010 | |  | |  | |  |
|  | **169** | 0.016 |  |  | |  |  | **86** | | 0.003 | |  | |  | |  |
|  | **170** | 0.001 |  |  | |  |  | **88** | | 0.004 | |  | |  | |  |
|  | **171** | 0.005 |  |  | |  |  | **90** | | 0.278 | |  | |  | |  |
|  | **172** | 0.033 |  |  | |  |  | **92** | | 0.557 | |  | |  | |  |
| **FE17** | **110** | 0.005 |  |  | |  |  | **94** | | 0.032 | |  | |  | |  |
|  | **116** | 0.833 |  |  | |  |  | **96** | | 0.060 | |  | |  | |  |
|  | **118** | 0.017 |  |  | |  |  | **98** | | 0.028 | |  | |  | |  |
|  | **120** | 0.145 |  |  | |  |  | **100** | | 0.011 | |  | |  | |  |
| **FE37** | **107** | 0.003 |  |  | |  |  | **102** | | 0.011 | |  | |  | |  |
|  | **109** | 0.223 |  |  | |  |  | **104** | | 0.001 | |  | |  | |  |
|  | **111** | 0.207 |  |  | |  | **D21** | **110** | | 0.003 | |  | |  | |  |
|  | **113** | 0.192 |  |  | |  | (*n* = 616) | **114** | | 0.015 | |  | |  | |  |
|  | **115** | 0.347 |  |  | |  |  | **170** | | 0.001 | |  | |  | |  |
|  | **119** | 0.028 |  |  | |  |  | **172** | | 0.567 | |  | |  | |  |
| **FE38** | **63** | 0.257 |  |  | |  |  | **174** | | 0.365 | |  | |  | |  |
|  | **65** | 0.258 |  |  | |  |  | **176** | | 0.049 | |  | |  | |  |
|  | **67** | 0.276 |  |  | |  | **De35** | **183** | | 0.001 | |  | |  | |  |
|  | **69** | 0.209 |  |  | |  | (*n* = 614) | **187** | | 0.147 | |  | |  | |  |
|  |  |  |  |  | |  |  | **189** | | 0.373 | |  | |  | |  |
|  |  |  |  |  | |  |  | **191** | | 0.039 | |  | |  | |  |
|  |  |  |  |  | |  |  | **193** | | 0.005 | |  | |  | |  |
|  |  |  |  |  | |  |  | **195** | | 0.005 | |  | |  | |  |
|  |  |  |  |  | |  |  | **197** | | 0.099 | |  | |  | |  |
|  |  |  |  |  | |  |  | **199** | | 0.037 | |  | |  | |  |
|  |  |  |  |  | |  |  | **201** | | 0.231 | |  | |  | |  |
|  |  |  |  |  | |  |  | **203** | | 0.044 | |  | |  | |  |
|  |  |  |  |  | |  |  | **205** | | 0.010 | |  | |  | |  |
|  |  |  |  |  | |  |  | **211** | | 0.009 | |  | |  | |  |

Ants (*Formica lugubris*, *n* = 547; squirrels (*Sciurus vulgaris*, *n* = 107), albatross (*Thalassarche melanophris*, *n* = 616), and kakī (*Himantopus novaezelandiae*, *n* = 98). Both the albatross and kakī dataset contained missing data for some loci, number of individuals genotyped at each locus is indicated below locus name. There were no missing data in the ant and squirrel datasets.
